# What GDPR and the Health Research Regulations (HRRs) mean for Ireland: a research perspective

**DOI:** 10.1007/s11845-020-02330-3

**Published:** 2020-07-29

**Authors:** Blanaid Mee, Mary Kirwan, Niamh Clarke, Aoife Tanaka, Lino Manaloto, Emma Halpin, Una Gibbons, Ann Cullen, Sarah McGarrigle, Elisabeth M Connolly, Kathleen Bennett, Eoin Gaffney, Ciaran Flanagan, Laura Tier, Richard Flavin, Noel G. McElvaney

**Affiliations:** 1 Department of Histopathology, St James’s Hospital, Dublin, Ireland; 2 Department of General Practice, Royal College of Surgeons in Ireland, Dublin, Ireland; 3 Department of Surgery, School of Medicine, Faculty of Health Sciences, Trinity College Dublin, Dublin, Ireland; 4 Biobank Ireland Trust, Dublin, Ireland; 5 School of Biotechnology, Faculty of Science and Health, Dublin City University, Dublin, Ireland; 6 Conway Institute of Biomolecular & Biomedical Research, University College Dublin, Dublin, Ireland; 7 Department of Surgery, St James’s Hospital, Dublin, Ireland; 8 Department of Epidemiology and Public Health Medicine, Royal College of Surgeons in Ireland, Dublin, Ireland; 9 Department of Medicine, Royal College of Surgeons in Ireland, Dublin, Ireland

**Keywords:** Consent, Data protection, GDPR, Irish Health Research Regulation, Patients & Data protection

## Abstract

**Background:**

Irish Health Research Regulations (HRRs) were introduced following the European Union (EU) General Data Protection Regulation (GDPR) in 2018. The HRRs described specific supplementary regulatory requirements for research regarding governance, processes and procedure that impact on several facets of research. The numerous problems that the HRRs and particularly “explicit consent” inadvertently created were presented under the auspices of the Irish Academy of Medical Sciences (IAMS) on November 25, 2019, at the Royal College of Surgeons in Ireland.

**Aims:**

The objective of this review was to obtain feedback and to examine the impact of GDPR and the HRRs on health research in Ireland in order to determine whether the preliminary feedback, presented at the IAMS meetings, was reflected at a national level.

**Methods:**

Individuals from the research community were invited to provide feedback on the impact, if any, of the HRRs on health research. Retrospective patient recruitment and consent outside a hospital setting for a multi-institutional Breast Predict study (funded by the Irish Cancer Society) were also analysed.

**Results:**

Feedback replicated the issues presented at the IAMS with additional concerns identified. Only 20% of the original target population (*n* = 1987) could be included in the Breast Predict study.

**Conclusions:**

Our results confirm that the HRRs have had a significantly negative impact on health research in Ireland. Urgent meaningful engagement between patient advocate groups, the research community and legislators would help ameliorate these impacts.

## Introduction

The European Union (EU) General Data Protection Regulation (GDPR) came into effect on May 25, 2018. GDPR permitted member states to introduce further protections or safeguards, with regard to personal data, including health data [1–3]. Ireland’s Health Research Regulations (HRRs) followed on August 8, 2018. The HRRs introduced additional regulatory requirements for health research in relation to governance, processes and procedures that impacted on several aspects of research [4, 5]. One of the requirements of the HRRs is that identified or identifiable personal data cannot be included in health research unless (a) explicit consent exists or (b) a consent declaration has been granted [1–7].

At a public meeting organised by the Irish Academy of Medical Sciences (IAMS), on November 25, 2019, GDPR was welcomed, yet the view was expressed that mandatory “explicit consent”, as imposed by the HRRs, was a significant impediment to conducting health research in Ireland.

Also presented were preliminary findings that the HRRs had precipitated [8] (a) huge site to site variation, because institutions had interpreted and applied the legislation differently; (b) serious concerns regarding Ireland’s ability to attract clinical trials now and into the future, due to site to site variability; (c) a go-slow in research, with some studies halted altogether due to (i) protracted decision-making on the part of institutions, (ii) immense outlay in both time and funds, required to implement GDPR and the HRRs, and (iii) difficulties navigating a new regulatory system for research.

It was felt that the common difficulties identified warranted further investigation. All feedback is presented herein. National feedback was sought to rule out institutional bias.

Lastly, an example of retrospective patient recruitment and consent for health research, outside a hospital setting, is included. The inclusion of this Breast Predict study serves as an example of the difficulties in:

1. Obtaining consent retrospectively outside a hospital setting, cost, staffing, response rate, attrition, patient capacity, establishing whether the patient is alive, emotional distress to patients and family members and understanding
2. The changing parameters around consent requirements and the difficulties posed to researchers
3. Divergence of opinions around consent requirements and the law
4. Lack of clear and consistent advice available to researchers
5. Difficulty applying the technical requirements of the HRRs in practice

## Methods

### Feedback from the research community on the HRRs

The purpose in obtaining feedback was to ascertain whether the preliminary feedback, presented at the IAMS November meeting, was reflected at a national level. This publication is not intended as an exhaustive assessment of Irish research post introduction of GDPR and the HRRs, but rather a collection of opinions from individuals attempting to navigate the new system, for the inclusion of data in research. Individuals were invited to provide information via email on any of the following: (a) general understanding of the HRRs, (b) time to obtain Research Ethics Committee (REC) approval, (c) RECs shutting down to become compliant, (d) RECs reluctance to accept external templates due to lack of nationally approved templates, e.g. patient information leaflets, (e) time outlay related to Health Research Consent Declaration Committee (HRCDC) applications and (f) Data Protection Officer (DPO) guidance. The established networks of the Irish Research Nurses Network (IRNN) and Cancer Trials Ireland were utilised to harness wider feedback.

### Irish Cancer Society (ICS) Breast Predict study: recruitment outside a hospital setting

The study “Towards personalised approaches to breast cancer treatment and prevention: establishing a retrospective breast cancer cohort” was ethically approved, on August 23, 2016.

This study sought to obtain consent retrospectively from previous breast cancer patients diagnosed at St. James’s Hospital between 2004 and 2014 to take part in a Breast Predict study and also consent to their inclusion in the St James’s Hospital Histopathology Biobank (SJHHB).

The Breast Predict Consent Form and Information Leaflet were modelled on the SJHHB documentation which underwent robust review, namely [9]:

i. Review by department of Legal and Insurance.
ii. Legal review from an independent medical lawyer.
iii. Review by the (then) Deputy Data Protection Commissioner, which focused particularly on the proposed method for data sharing.
iv. Review by patient advocate group—Europa Donna Ireland.
v. NALA (National Adult Literacy Agency) review—NALA translated the patient information leaflet (PIL) into “plain English”, ensuring that the document was simple and easy to follow.
vi. Review by hospital research and ethics committee.
vii. In 2018, a review of SJHHB documentation was conducted by an independent and unbiased Data Protection Consultant. Their report defined the description of “Future Research and Studies” on the consent form, as “good”, and the purpose, as “specific”.

It was decided that a research nurse (RN) was best placed to (a) contact patients and next of kin, (b) handle questions posed by respondents, (c) understand concurrent illnesses capable of impairing an individual’s capacity to consent, (d) liaise with clinical teams, where necessary. The RN was employed for a fixed term of 12 months at a cost of almost €30,000 (19.5 h per week). An introductory letter, detailing the objectives of the study, and consent form were posted. A prepaid envelope provided the means of returning the consent forms.

## Results and discussion

### Feedback on the HRRs

Respondents echoed feedback presented at the November IAMS public meeting [8]. Additional concerns were (a) the absence of meaningful consultation when drafting the HRRs; (b) difficulty comprehending the HRRs and confusion regarding their impact on clinical trials; (c) the requirement for *Ireland only* amendments to international clinical trial documentation, to reach the threshold of “explicit consent”, required by the HRRs; (d) the future of non-interventional clinical trials and critical care research in Ireland; (f) the negative impact on PhD (Doctor of Philosophy), MD (Doctor of Medicine) and ICAT (Irish Clinical Academic Training) programmes; (g) Ireland’s capacity to contribute to large international collaborative research projects; and (h) reports that the increased bureaucratic burden (ethical and HRRs related) had slowed and in some cases halted research (see Table 1).

**Table 1:** Quotes detailing the impact of the HRRs on health research*

Respondent	Feedback
Principal investigator	“Those of us who work in clinical research understand the need for regulation and adherence to best practice. While improvements in regulation are always welcome, the introduction of GDPR and HRRs have significantly hampered Irish research, in particular the decision to retrospectively apply a new standard to established studies. The shifting sands of GDPR and HRRs interpretation has resulted in a lack of clear communication of what is required from both researchers and patients. In the absence of clear guidance from the Department of Health (DoH), hospitals have defaulted to an overly stringent interpretation in order to avoid legal issues. In the 18 months since GDPR there has been stasis, uncertainty, frustration and a lack of clear and consistent direction as how to implement these regulations. These issues have caused a huge impact on Irish research. Pre GDPR studies have stalled or prematurely ended as the funding deadlines pass while decisions are still awaited on whether documentation is now compliant. For new studies, patients are now bombarded by lengthy, legalistic consent forms that serve to undermine the principles of informed consent. As a researcher, I have made difficult decisions not to pursue a number of international collaborations as transferring of coded clinical data outside the EU as the process for how this can proceed is uncertain and the paperwork now virtually requires a team of administrative staff. Central to the issues with the new regulations is that much successful Irish research is Investigator led and patient focused. The complexity and uncertainty introduced with the new regulations is actively deterring Irish researchers from entering or continuing in research. Many of us juggle research with clinical posts, we perform research as we are committed to improving patient care. In the era of the HRRs, research will be ever more restricted to those with large administrative teams (such as pharmaceutical companies) for whom profits rather than patients are the driving force. The unintended consequences of HRRs and GDPR are multiple but often forgotten is the burden on our patients. The current complexity of consent and the re-contact of patients on previous studies is actively dissuading patients from research participation. The restrictions on who can screen for inclusion has resulted in patients denied opportunities for involvement in research that could be of benefit to them. These factors are further compounded by the lack of decision making centrally, with little progress seen in the last 18 months. Our patients deserve better”.
Anonymous DPO	“The extra safeguard of explicit consent introduced by the HRRs, caused utter confusion amongst DPOs. DPOs (many new to their roles) were the arbitrators of whether the consent obtained for ongoing research was sufficient to comply with the HRRs, or whether an application to the HRCDC was required. The practicalities of obtaining consent for pre-screening and retrospective chart review were not considered in the HRRs. The amendments to rectify this oversight, have taken far too long and in the meantime (over one year) retrospective chart review research came to a complete standstill……. Ongoing research also came to a standstill prior to HRCDC applications as it was not clear whether such research could continue or not…..”
Professor Mark Little, Trinity College Dublin	“Over the past year, we have diverted most of our resources to aligning with the health research regulations. Research has had to take a back seat. We could not have re-booted a HRRs compliant registry and biobank without close and ongoing support from our local Clinical Research Facility (CRF) regulatory affairs team; this cost us €17,000 and counting, money which has been diverted away from research”.
Professor William Gallagher, University College Dublin	“I really wonder sometimes how much the patient community has been consulted and listened to in the efforts to implement the apparently overly strict regulations that we now seem to be facing in terms of GDPR, especially in the context of health research. Particularly in the cancer space, which I know best, it is clear that most, if not practically all, patients who volunteer their own tissue materials and other biological samples for research do so with a view to advance research in a general way. The nature of scientific exploration often throws up new opportunities and ideas, which were not or could not be foreseen in advance. A flexible and pragmatic approach should be taken to ensure that maximal use is gained from the precious materials kindly donated by patients”.
Research nurse	“I have worked in clinical research as a coordinator for clinical trials, for many years and I have always been able to rely on my local Ethics committee for an informed and comprehensive response to any questions I might have about a study. It’s a different story since GDPR, because the goal posts seem to change on an almost weekly basis regarding what is and is not allowed. Can we approach patients or do we need explicit consent? How do we inform patients about studies that they may potentially benefit from and still adhere to GDPR? Do we have to re-consent retrospectively, on studies started prior to GDPR? So many questions and so little clarity on how best to proceed. This can only have a negative effect on Clinical Research for Ireland, if a Sponsor for a Clinical Trial has to wade through, complex and unclear regulation surrounding GDPR, then who can blame them for going elsewhere, to another country, where these issues have been clarified and the pathway is well defined”.
Research Ethics Committee Coordinator	“Where would we be if there had been GDPR only, and no Health Research Regulations? What would have happened if the Department of Health and Health Research Board had spent the 2 years between May 2016 and May 2018 preparing the research community for GDPR? What would have happened if the Department of Health and Health Research Board had indicated to the research community they were considering availing of the option within GDPR for member states to introduce their own legislation around health research? What would have happened if the Department of Health and Health Research Board had consulted with the research community and engaged in a debate about what national legislation would look like? Would the Health Research Regulations have been signed? Would the date of enactment have been the same? Would forewarning and time to prepare have helped? Might the content have been different? As it happened, the Department and Health Research Board decided not to inform, forewarn, consult or debate; they drafted in secret, signed into law suddenly, and blindsided the research community. The amount of confusion the Health Research Regulations have caused cannot be understated. The amount of work for every individual in the research community to try to understand the legislation and attempt to comply with it has been crushing. The stress and mayhem created by the Health Research Regulations cannot be put into words. The Department of Health and Health Research Board must never be allowed to do this again. Amendments to the legislation are not enough. Nothing less than repeal and redraft is acceptable. For this to occur, the Department of Health and Health Research Board need to see the impact this legislation has had. I would like to thank the authors for their bravery in writing this paper”.
Anonymous researcher	“We are a small research team (2 people) offering many services on a shoestring. These include information, diagnosis, research, and advocacy services as well as co-ordinating a national rare disease registry which has over 600 participants. If we were forced under the HRRs to attempt to re-consent, these vital services would grind to a halt. And there is nobody else working on this rare disease in Ireland. Getting ready for GDPR and then applying for an exemption for the registry has already cost us huge time and effort. We remain in limbo waiting for a decision on this. We are not Facebook or Google. Our motivation is better health and the registry is a vital recruitment tool for clinical trials testing new, potentially life-changing treatments, with several starting in 2020. Will the registry participants be denied the chance to participate if we cannot obtain an exemption?”
Irish Research Nurse Network Feedback	“GDPR itself has not negatively impacted on research but the Health Research Regulations have been harmful to research opportunities and collaborations. Part of being a student in healthcare (regardless of discipline) is learning about research, how to conduct and interpret etc. There are many reports and papers that show how research active units and research aware staff improve patient outcomes. This is recognized by the DoH with the ICAT Programme (this is just one example). However, with the HRRs and need for explicit consent, updating consent forms etc it meant that where students would previously been offered a summer placement or opportunity to participate in research has now been withdrawn in a lot of cases. One example was the HRB Summer Scholarship Scheme. As a Research Coordinator (in 2018) I had projects ready for students (most involved gathering data, retrospective, from charts). Students would be involved from deciding what data points to collect to doing some of the analysis. Due to the uncertainty surrounding the HRRs and need for consent all but one student decided to pursue a Health Research Board (HRB) Summer Scholarship. This meant that research projects, to improve patient care, were not completed and students missed an opportunity to learn about how to determine what data is needed, how to collect and store it, analyse it, interpret it, put the results into bedside care etc. Studies, that meet the consent requirements at the time, and were international collaborations were put on hold. This impacted on our unit’s ability, on an international stage, to participate and be involved in the development of studies in a specialized area of care. This will have long term impacts as Ireland as a whole is showing that is not research friendly and many other research/healthcare centres etc will go to other countries to collaborate on studies. I was very surprised by the lack of awareness by Clinical Trial and Regulatory Affairs/Quality Managers on the requirements of GDPR and HRRs. For example, under Article 3 of GDPR (Territorial scope) and the need for contractual standard clauses when transferring data outside of the EU or need to gain the consent of children once they reach 16 years if we wished to continue to gather follow-up data from paediatric trials that they had participated in. One manager felt that clinical trials were governed by the Clinical Trials Directive and therefore GPDR/ HRRs did not apply! It caused tension with the sponsors of clinical trials as they kept saying they were GPDR compliant and we had to explain to them about the layered requirements as set out by the HRRs (which is only applicable in Ireland). This meant that sponsors had to develop new versions of consent forms etc just for Ireland. As we are a paediatric hospital, this meant that sponsors were undertaking additional work for one site only. Also noticed that industry interest in perusing clinical trials in our area decreased after the Health Research Regulations were brought in”.
Professor Ger Curley, Professor of Anaesthesia and Critical Care	“I lead a research program in critical care, including basic biomedical studies, observational studies and clinical trials. The Health Research Regulations stopped our research work completely for almost a year. Now, we are significantly curtailed. We cannot use the data stored in our Clinical Information System, one of the first electronic medical records in Ireland. Lessons we could potentially learn from this huge store of data are closed off to us. The approval process required in order for us to recruit patients into studies in ICU takes such a length of time that other sites in other countries have completed the studies before we can join. Students who wish to do ICU research will potentially spend the first year of PhD or MD programs applying for approval to conduct studies. These obstacles are making our research program unattractive for students, for collaborators and for industry. And ultimately, patients will suffer, as they will not be able to access potentially lifesaving treatments”.
Professor Gianpiero Cavalleri, Royal College of Surgeons, Ireland	“I think everyone wants the same thing – for effective research to be carried out in a way that balances the health of the population with individual privacy rights. But we need to collectively and quickly evolve a system that truly serves this purpose, and is informed by all stakeholders. From the researcher perspective, there is too much bureaucracy and uncertainty with the current system. This is slowing the science, which is designed to promote the health and wellbeing of society”.
Postdoctoral researcher, Trinity College Dublin	“These regulations have made it extremely difficult for researchers to conduct any actual research over the last few years, with our time and efforts being diverted to keeping up with constantly-changing guidelines and paperwork, and ‘training sessions’ where there are more questions than answers. Working between a hospital and a university has been particularly challenging, as no clear or unified guidelines have yet emerged from either institution. Our department researches a rarer cancer type and pre-cancerous conditions. We therefore rely heavily on access to archival specimens and data to carry out research on large enough cohorts, and therefore specific, ‘granular’ consent is not possible unless patients are re-contacted and re-consented every time a researcher wants to use their specimens or data. We do not have a reliable means of finding out whether a patient has died, meaning we run the risk of contacting and upsetting bereaved families. While we do see the need for regulation in data protection, we do not currently have the infrastructure or suitably qualified personnel available to carry out a re-consenting process, and no funding to support this has been forthcoming. Many current projects are essentially on hold, until we get clarification on whether we can proceed with things like third party sharing for collaborative work. This has had a knock-on effect and has stalled my lab’s research publication output, which will badly affect my future employment and grant funding prospects as an early career Principal Investigator. We have made an application to the HRCDC detailing these issues in the hope that we can get a clarification on how to proceed, but it is likely I will not still be employed as a researcher by the time a decision is made, since all my grants are ending soon. I am sure other people have been lost to research as a result of this working environment. The worst part of this issue is that the public and patients seem to be unaware that research is currently being restricted in this manner. From my outreach work with patients, I feel that a more sensible arrangement could be worked out if this issue was brought to the main stakeholders, including us staff on the ground. That these impractical regulations have been implemented without proper consultation is particularly egregious”.
Professor Aurelie Fabre, Consultant Histopathologist	“It is impossible to re-consent everyone who has already consented to research and given samples to biobanks. Often finding out if the person is alive or dead is a challenge. Patients who have already given consent for research believe that their samples and/or data will be used for the greater good. For rare disease research this is even more important as there are very few archival samples. Finally, the trust and commitment of research funders and their contributors (taxpayers and donors to charities) is being jeopardised as research is significantly delayed or stopped (e.g. researchers left in limbo waiting for consent committee decisions)”.
Professor National University Ireland Galway	“GDPR brings welcome increases in data security and protection for citizens, organisations and governments. In the main, GDPR reinforces current best practices in research. However, implementation of GDPR has consequences not least of which is the requirement for greater resourcing of legal advice specific to GDPR to researchers”.
Feedback from three SpRs (specialist registrars) working in different disciplines	“There are numerous factors detracting NCHDs from completing their training in Ireland. NCHDs are exposed to a challenging work environment, with extensive duress placed upon junior doctors to provide a functional health service with limited reward. Internationally junior doctors have protected teaching time, allocated study days and allotted research commitments. On paper, these training requirements are documented requirements for junior doctors in Ireland. On the front line of healthcare in Irish hospitals however, the daily practicalities of such is non-existent. Junior doctors struggle to obtain the required days off to attend training events, attend conferences or conduct meaningful research. With the new legal framework of GDPR, especially regarding the interpretation in Ireland, this has pressed added demands on junior doctors and has averted trainees from engaging in research. Firstly, Irish doctors rotate through varying hospitals with maximum time spent in one institution for most trainees being one year. Due to the backlog of requests for REC presently in place, junior doctors are forced to wait up to 6 months for their proposed projects to be reviewed. From time of initial project development in the brainstorming period, to writing the ethical approval, the data protection impact assessment form, the patient information leaflet and the consent form, this may take weeks. With the addition of time to implement any amendments the REC or the respective DPO, junior doctors will be rotating out of their respective hospital to new sites. Thus, leaving projects unfinished. Furthermore, if numerous sites are involved for a research project, separate ethical approval forms, the data protection impact assessment forms, the patient information leaflet and consent forms are required for each institution. The extensive paperwork required varies. For example, my latest research proposal included the following: Ethics application: 12483 words. 41 pages DPIA: 8369 words and 35 pages Patient Information leaflet: 2804 words and 12 pages Consent Form: 380 words and 3 pages This was just for one institution. For the remaining institutions I had to complete separate forms for each. It has been my experience that consultants themselves are very aware of the GDPR issue and the limitations that are there for NCHDs conducting research in Ireland. However, consultants are themselves disillusioned in conducting research in Ireland and thus not putting themselves forward as potential supervisors for research projects. From discussions with fellow NCHDs, the viewpoint has been to see their higher specialist training in Ireland to completion and then once on an international fellowship, to complete any further research commitments there. With the limitations in place in Ireland and constraints placed on getting proposals reviewed at RECs, NCHDs are leaning away from research in Ireland. The varying viewpoints of the REC’s interpretation of the legislation are also placing doubts in researchers’ minds. The confusion that appears to be present regarding the varying stances that different RECs in varying institutions are stating, is also deterring junior doctors from engaging in research in Ireland. NCHDs have heeded advice to restrict research to deceased patients given that they fall out of the GDPR remit. This may throw up further issues, as will research in Ireland be limited to this cohort for junior doctors to circumvent arduous delays in REC wait lists, avoid having to complete further extensive paperwork, with hope to churn out research projects/ scientific papers in order to further their knowledge and training. Combining research with your day job is essential for progression for junior doctors, and we usually get through it in snatched hours in the evenings after work. The era of GDPR, and the extraordinary demands it makes, have ground this almost to a halt. The legislation was aimed at huge companies that can afford entire legal departments, but I feel like scientists and doctors have become its collateral damage. Now, before we can even open a chart for a retrospective review, we need to complete a Data Protection Impact Assessment (DPIA) to explain what ‘consultation process’ we have arranged to ‘consult with stakeholders’. It is full of jargon that is poorly understood (‘special category data’ and ‘function creep’ leap out). My last DPIA was 8 pages long and required references to various legal articles within the GDPR regulation text that no lay person would understand. The subsequent ethics proposal asks many of the same questions and is 25 pages long. Once all of these are gathered, your ethical approval may take months to be considered and rejected over minor details. In the context of jobs that last a couple of months or a year at maximum, many doctors I know no longer see small research projects as a realistic prospect and are simply not taking them on. Those that do are struggling to get anything done. Those of us who are sub-investigators in larger projects spend a lot of time re-consenting patients due to minor changes in study protocol. GDPR has been a really frustrating experience for us and it is hard to see what benefits come to society from such intensive policing of small projects”.
Eibhlín Mulroe, CEO, Clinical Trials Ireland	“Since the arrival of GDPR, contract and ethical processes are stalled to slo-mo at some of our hospital sites. We are seeing a significant impact on the numbers of patients recruited to trials at sites because of varying interpretations of the law – access to trials is becoming a postcode lottery”.
Patient advocate	“A huge number of the challenges that have arisen since the introduction of the HRRs have stemmed from communication issues. These issues have included: • a failure to truly consult with researchers and patients prior to the introduction of the HRRs • an unwillingness by some within the research community to recognise the perspectives and restrictions of the legislators • a lack of any group with sufficient resourcing to provide strong, consistent guidance on the interpretation of the legislation from the outset. Amendments proposed by the Department of Health, the useful information now being provided by the HRCDC and a plan for an increased role in communication by the DPC should all help in the slow journey towards overcoming the challenges and help with a healing of rifts. We know from our nearest neighbours on either side how detrimental the polarisation of society can be. For the sake of patients, we need to work together better to figure all this out”.

*All feedback is presented herein unaltered

### Breast Predict—retrospective recruitment and consent outside a hospital setting

As per the Guide to Professional Conduct and Ethics for Registered Medical Practitioners, the Tallaght University Hospital/St. James’s Hospital (SJH) Joint Research Ethics Committee deemed that consent from the next of kin was required where possible, where patients had died [10]. The pathway for contact and consent was decided after discussions between clinicians, biobank staff and the legal team. The inclusion of GPs (general practitioners) as gatekeeper to contacting patients or their next of kin was important in attempting to establish whether clinical changes had occurred in the interim, which might affect the participants’ capacity to consent. Over 1 year, 812 patients and next of kin were contacted (Table 2). On 104 occasions, GPs advised against contact. Reasons included dementia, patient in long-term residential care and grief, or anger, at patients passing. Six hundred and fifteen (76%) of those contacted responded. Quality control of returned consent forms led to the exclusion of 22% of respondents, due to inaccurate or partial completion of forms. The section most commonly not initialled related to financial gain, i.e. *I understand I will not make money if research leads to a new test or treatment*. It was difficult to interpret the respondents’ opinion or concerns around “financial gain”. Did they believe that research in an academic environment is preferable to research performed by a commercial entity? This was complex to evaluate, as one of the principal aims of health research must be the identification of translational elements, which may lead to the development and delivery of novel tests or treatments, for patients. Delivering such innovation, without industrial partners, would be impossible.

**Table 2:** Recruitment outside a hospital setting for the Breast Predict study

Total number of participants in study	1987
Total number of letters introducing study sent to GPs	673*
Undelivered letters returned from GPs:	360 (53.5% of GPs)
Breakdown of responses provided by GPS:	
1. Patient(s) and NOK (next of kin) both deceased	15
2. Patient(s) and NOK alive but contact not advised due to impairment	38
3. Patient(s) deceased (contact with NOK not advised due to impairment) or absence of contact information (NOK not patient of GP)	19
4. Patient(s) deceased—no NOK contact	149
5. Patient(s) moved GP and no current contact details	36
6. Patient(s) unknown to GP	22
Total number of incidences where GP advised against contact:	104
Reasons provided by GP: i) Dementia ii) In long-term residential care iii) Distressed NOK due to relatives’ or spouses’ death	
Letters re-sent to GPs	68
Letters sent to patients/NOK:	812 (40.9% of all participants)
Total number of returned form (yes, no and incomplete responses)	615 (75.7%)
Letters re-sent to patient/NOK:	304 (37.4%)
Initial assessment of number (patient/NOK) happy to partake:	509 (62.7% of returned forms)
Revised number (patients/NOK) happy to partake, *post quality control review of returned consent forms*:	399 (49.1% of returned forms)
Patient/NOK excluded from study	216
Reasons for exclusion	
i) Patient/next of kin ticked “no” (to research)	48
ii) Blank introductory letters returned (interpreted as “no”)	17
iii) Blank consent forms returned (interpreted as “no”)	17
iv) Consent form returned with sections ticked, *not* initialled	1
v) Consent form returned with signature; however individual sections on the consent form *not* initialled and “I do not consent” box *not* ticked: lack of clarity	55
vi) Incomplete consent forms (1 or more sections *not* initialled)	78

*Several GPs were contacted with regard to one or more patient(s)

Tiered consent, though valuable, inadvertently created an “all or nothing situation”, that is unless a patient, or next of kin, consented to all sections of the consent form; it was difficult to manage the inclusion of associated samples and data. From a legal perspective, especially that of “explicit consent”, it was safer to simply omit incomplete consent forms. Some of the one hundred and ten respondents excluded post quality control could potentially have been included, subsequent to follow-up. However, several issues prevented this recontact: (a) The RN’s contract had expired and the biobank lacked resources to perform this role. (b) It was unclear what level of “follow-up” would be satisfactory. It is not clear whether a written record of telephone conversations, where respondents clarified their wishes, would have satisfied the requirements of “explicit consent”. (c) The prospect of recontacting patients or their next of kin, for a second time, raised concerns. Specifically, the potential stress this may cause. It was unclear how to strike a balance to ensure the wishes of patients and their next of kin were upheld with that of an individual’s right to privacy. For example, an acceptable frequency of telephone calls and written communication could not be established.

In total, 399 patients’ samples out of a potential 1987 could be included in the study (see Table 2).This represented just 20% of the original study target. Managing tiered consent, with multiple choices, in the absence of a Biobank Information Management System (BIMS) also proved extremely difficult and costly in terms of time.

During the period of retrospective consent (October 2017 to October 2018), three distinct laws were in place: (a) Data Protection Acts 1988 and 2003, (b) GDPR and (c) GDPR and the HRRs. GDPR and the HRRs were respectively introduced at 6 and 10 months into the process [1–7]. Towards the end of the research nurse’s contract, it became unclear whether the retrospective consent was GDPR and HRR compliant and even were funds available, whether it would be ethical to return to patients and next of kin again. Many questions remain, namely, (i) what constitutes compliant documentation, (ii) what constitutes sufficient transparency in terms of future use of data and (iii) whether a HRCDC application is required. This is discussed in detail in the companion paper; What GDPR and the Health Research Regulations (HRRs) mean for Ireland: “explicit consent”—a legal analysis.

Participant consent has long existed as an ethical and legal requirement for the conduct of health research in Ireland [11, 12]. Therefore, the concept of “explicit consent”, as required by the HRRs, may seem reasonable. However, as a legal instrument, the prerequisite of participant “explicit consent”, as presented within the HRRs, neither encourages nor facilitates health research [3, 13]. The Patient Voice in Cancer Workshop sought to understand what information was most pertinent to participants [14]. Crucial given the level of information, researchers were encouraged to furnish participants with, in order to achieve “explicit consent” [15]. Importantly for re-consent, patients viewed information leaflets and consent forms as “companion documents”, i.e. they assumed someone would sit with patients and go through the documents [14]. Though this adds to the complexity of retrospective consent, it reinforces the need for simple, clear and patient-friendly documentation.

## Conclusion

The findings presented at the IAMS on November 25, 2019, at the Royal College of Surgeons, Ireland, are upheld.

The introduction of legislation and standards to afford greater protection to health research participants will always be welcome. However, legislation and standards are only of value when achievable. Research infrastructure has never been prioritised in Ireland. Therefore, legislators and funding agencies must furnish the research community with the infrastructure required to achieve compliance and attain standards [16].

It is important to state that the intention of the legislators was not, in the authors’ opinion, to impede health research. Rather GDPR presented a mechanism to regulate research and simultaneously afford greater autonomy to health research participants. Inadvertently, however, the HRRs have heavily impacted on Ireland’s capacity to conduct health research, including clinical trials (both interventional and non-interventional) and caused significant damage to Irish research. It is hoped, that the findings presented herein provide sufficient grounds to prompt a review of the legislation.

The authors seek the regulation of consent in health research to a high standard. Getting data protection right in health research is important and vital to ensuring public trust of and continued support of research.

## Recommendations

1. Several proposed amendments were presented at both IAMS events in an attempt to rectify some of the problems created by the HRRs. Despite this these amendments have not been enacted, and there has been no attempt by the Department of Health to set up a consultative process with IAMS to discuss these proposals. This is an obvious area which needs to be addressed.
2. There must be an urgent review of the HRRs GDPR “explicit consent” requirement.
3. The regulation of informed consent in research in line with the common law and the approach taken by other EU member states.
4. Meaningful dialogue and consultation between legislators and key stakeholders including IPPOSI (Irish platform for Patient Organisations, Science and Industry), patient advocate groups and the research community are urgently needed.
